# Ion channels in control of pancreatic stellate cell migration

**DOI:** 10.18632/oncotarget.13647

**Published:** 2016-11-26

**Authors:** Hannah Storck, Benedikt Hild, Sandra Schimmelpfennig, Sarah Sargin, Nikolaj Nielsen, Angela Zaccagnino, Thomas Budde, Ivana Novak, Holger Kalthoff, Albrecht Schwab

**Affiliations:** ^1^ Institut für Physiologie II, 48149 Münster, Gemany; ^2^ Institut für Physiologie I, 48149 Münster, Gemany; ^3^ Section for Cell Biology and Physiology, Department of Biology, University of Copenhagen, DK 2100 Copenhagen, Denmark; ^4^ UKSH, Campus Kiel, Institut für Experimentelle Tumorforschung (IET), Sektion Molekulare Onkologie, D-24105 Kiel, Germany

**Keywords:** pancreatic stellate cell, migration, K_Ca_3.1 channel, TRPC3 channel

## Abstract

Pancreatic stellate cells (PSCs) play a critical role in the progression of pancreatic ductal adenocarcinoma (PDAC). Once activated, PSCs support proliferation and metastasis of carcinoma cells. PSCs even co-metastasise with carcinoma cells. This requires the ability of PSCs to migrate. In recent years, it has been established that almost all “hallmarks of cancer” such as proliferation or migration/invasion also rely on the expression and function of ion channels. So far, there is only very limited information about the function of ion channels in PSCs. Yet, there is growing evidence that ion channels in stromal cells also contribute to tumor progression. Here we investigated the function of K_Ca_3.1 channels in PSCs. K_Ca_3.1 channels are also found in many tumor cells of different origin. We revealed the functional expression of K_Ca_3.1 channels by means of Western blot, immunofluorescence and patch clamp analysis. The impact of K_Ca_3.1 channel activity on PSC function was determined with live-cell imaging and by measuring the intracellular Ca2^+^ concentration ([Ca^2+^]_i_). K_Ca_3.1 channel blockade or knockout prevents the stimulation of PSC migration and chemotaxis by reducing the [Ca^2+^]_i_ and calpain activity. K_Ca_3.1 channels functionally cooperate with TRPC3 channels that are upregulated in PDAC stroma. Knockdown of TRPC3 channels largely abolishes the impact of K_Ca_3.1 channels on PSC migration. In summary, our results clearly show that ion channels are crucial players in PSC physiology and pathophysiology.

## INTRODUCTION

Pancreatic stellate cells (PSCs) constitute a small fraction of cells in the periacinar space that are normally quiescent. Their function in a healthy pancreas has begun to be unravelled only recently. Roles in matrix turnover, regulation of exocrine secretion, immune functions, as well as progenitor functions have been shown [1–3]. It is well established that activated PSCs as well as infiltrating myeloid cells and lymphocytes are of great importance in pancreatic pathologies such as chronic (alcoholic) pancreatitis or pancreatic ductal adenocarcinoma (PDAC). PSCs are the predominant mesenchymal cells within the PDAC stroma [4]. They are activated by growth factors and cytokines (e.g. PDGF and TGF-β, IL8 and TNF-a) produced by tumor cells or by themselves (autocrine stimulation [5, 6]). Activated PSCs have an enhanced migratory activity, and in turn, secrete growth factors and cytokines. In PDAC this is the basis for mutual paracrine stimulation of tumor and stroma cells that leads, in a positive feedback cycle, to excessive matrix production (desmoplasia) and creates a hypoxic microenvironment conducive to invasive tumor growth and metastasis [7, 8]. Thus, the characteristic tumor stroma of PDAC and the mutual interaction of all its components play important roles in many aspects of tumor progression and in preventing efficient therapy. Accordingly, activated PSCs are in part responsible for the high mortality of PDAC patients whose 5-year survival rate is less than 5% [9]. However, there is some controversy with respect to this issue, since myofibroblasts have recently been shown to protect the host from the tumor [10, 11].

So far, there is limited knowledge on the function and expression of ion channels in PDAC [12–17]. Based on the critical role of ion channels in other tumors [18], it is reasonable to predict that they are important drivers of PDAC progression as well. We assume that this also applies for ion channels in PSCs. However, so far the role of ion channels in PSC physiology and pathophysiology is rather unexplored. There are only very few studies addressing this topic [19–22] some of which were performed on the closely related hepatic stellate cells [23, 24]. Yet, in analogy to other cell types of the tumor stroma it is very likely that altered ion channel expression and function in stimulated PSCs contribute to processes that are critical for PDAC progression such as cell migration and invasion, growth factor signaling, or proliferation and apoptosis [25]. In the present study we investigated the role of ion channels in PSC migration. Migration of PSCs has to be seen against the background that PSCs are also found in distant PDAC metastases [2] so that migration of PSCs is one of the prerequisites of co-metastases [25]. One of the best studied ion channels involved in cell migration is a Ca^2+^ sensitive K^+^ channel of intermediate conductance, K_Ca_3.1, which is expressed in almost all migrating cells [26–28]. Here we show that K_Ca_3.1 channels are also expressed in PSCs and contribute in cooperation with TRPC3 channels to their migratory activity.

## RESULTS

### Functional K_Ca_3.1 channel expression in RLT-PSC cells

**Figure 1:**
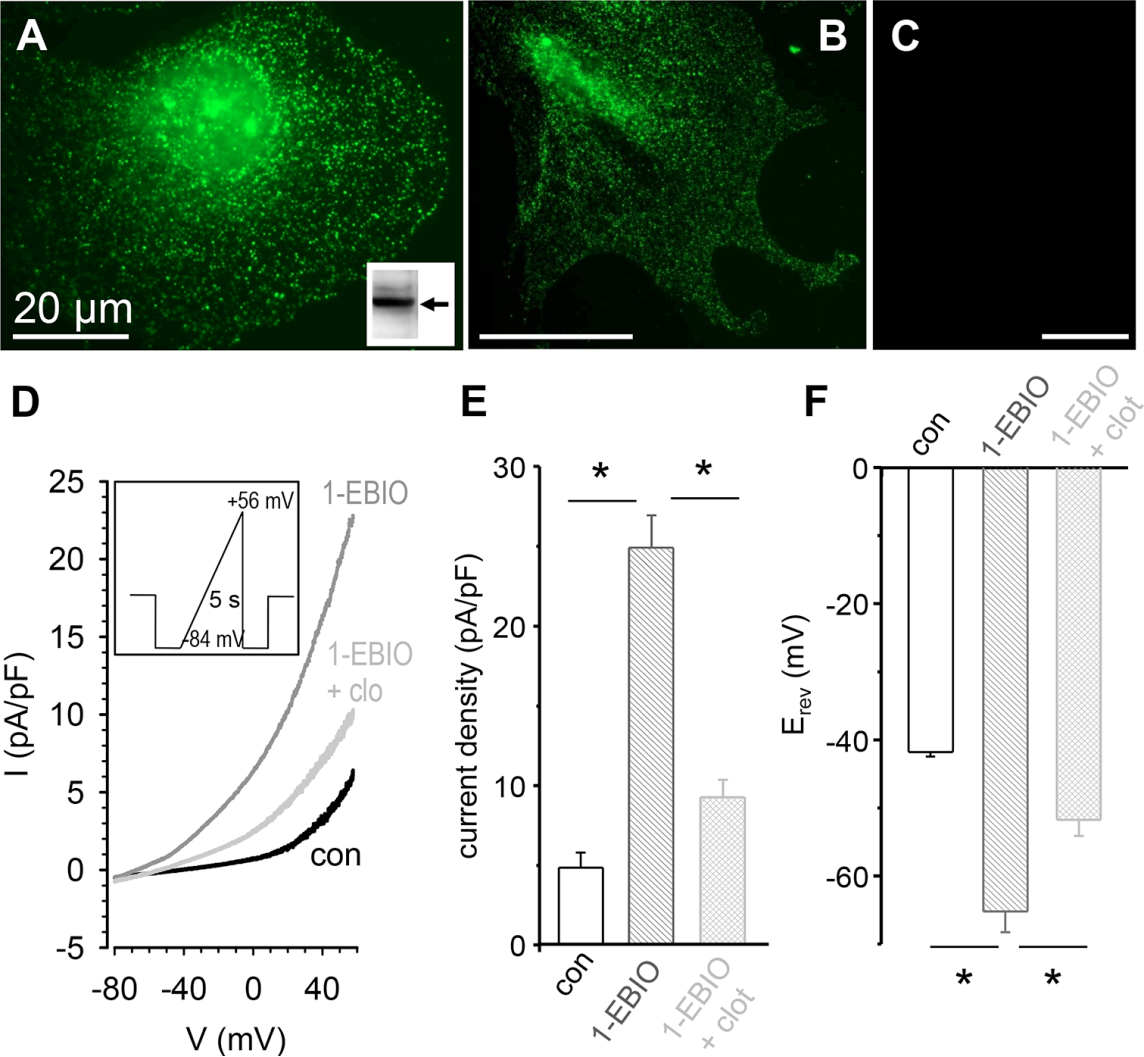
Expression of KCa3.1 in RLT-PSCs (**A**, **B**) Immunofluorescence and Western blot. Staining of K_Ca_3.1 channels in RLT-PSCs (A) and primary murine PSCs (B) by indirect immunofluorescence reveals the typical punctate pattern. Inset: Western blot analysis yields a band of the expected size (~50 kD). (**C**) K_Ca_3.1 channels are not detected in PSCs from K_Ca_3.1^−/−^ mice. (**D**) Original recording of a patch clamp experiment in the whole-cell configuration. The holding potential was −40 mV. We applied a voltage ramp of 5 s duration from −84 mV + 56 mV. The K_Ca_3.1 channel activator 1-EBIO (50 μmol/l) produced a large outward current which was inhibited by clotrimazole (1 μmol/l). (**E**, **F**) Summary of the patch clamp experiments. The current densities (pA/pF) are plotted in E., and F. depicts the reversal potentials (*n* = 9). * denotes *p* ≤ 0.05.

Western blot analysis revealed that K_Ca_3.1 channels are expressed in RLT-PSCs. Incubation of RLT-PSCs in conditioned PDAC cell medium does not increase K_Ca_3.1 expression, which is consistent with the analysis of published micorarrays (see below). Immunofluorescence staining yielded the typical punctate staining of the channel protein (Figure 1A). A similar staining pattern is also observed in primary cultures of murine PSCs (Figure 1B). K_Ca_3.1 staining is absent in PSCs isolated from K_Ca_3.1^−/−^ mice (Figure 1C). The final proof for the expression of functional K_Ca_3.1 channel proteins in the plasma membrane was obtained from patch clamp experiments. Using a KCl containing pipette solution we recorded an outwardly rectifying whole-cell current with the typical pharmacological properties of K_Ca_3.1 channels (*n* = 8; see Figure 1D [29]). Mean current density rises from 4.8 ± 1.0 pA/pF under control conditions to 24.9 ± 2.0 pA/pF in the presence of 50 μmol/l 1-EBIO. Clotrimazole (1 μmol/l) reduces current density to 9.3 ± 1.1 pA/pF in the continued presence of 1-EBIO (Figure 1E; *n* = 9). The respective reversal potentials are −41.8 ± 0.7 mV (control), −65.2 ± 3.0 mV (1-EBIO), and −51.7 ± 2.4 mV (1-EBIO and clotrimazole) (Figure 1F), which is consistent with the activation and subsequent (partial) inhibition of a K^+^ current.

### Stimulation of migration of PSCs requires K_Ca_3.1 channels

**Figure 2:**
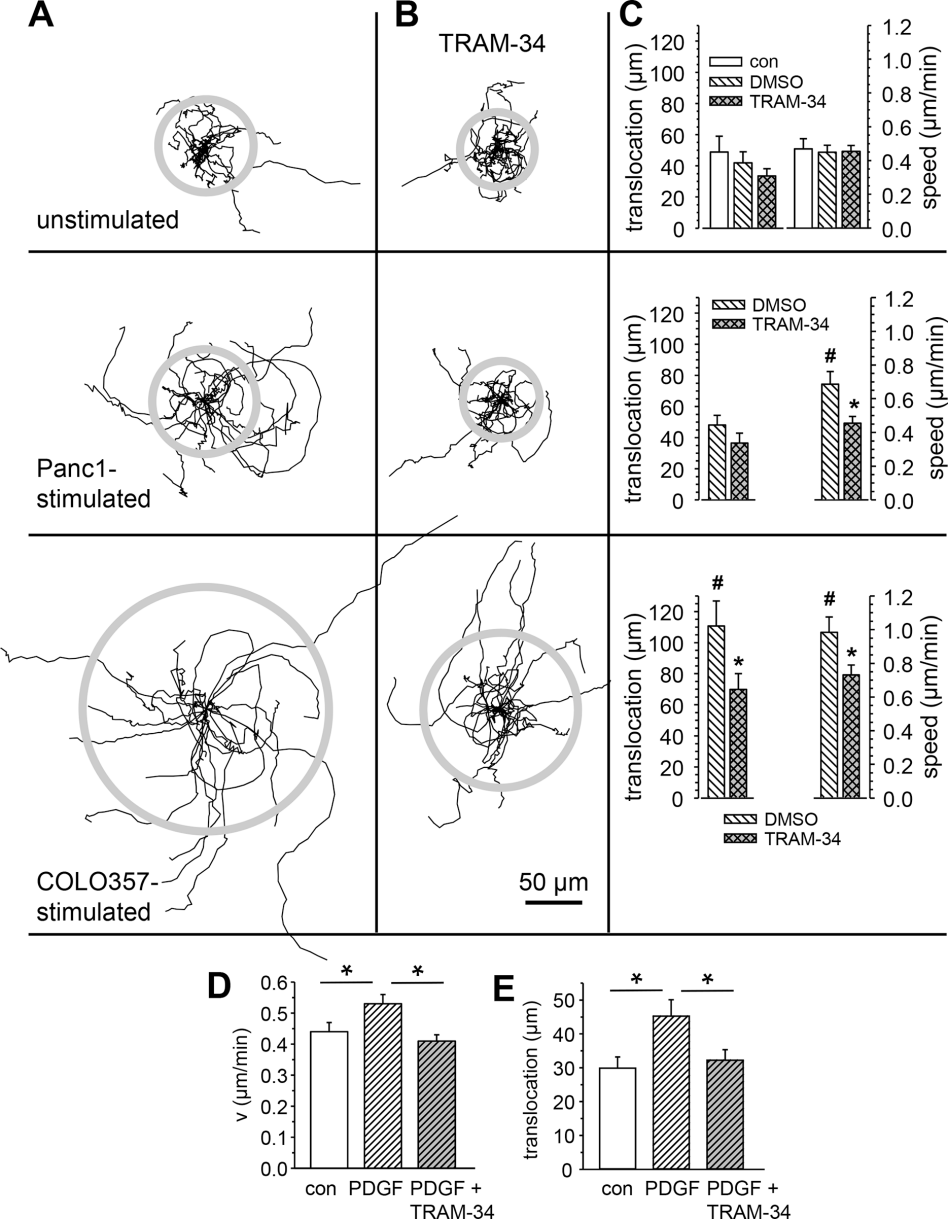
Stimulation of RLT-PSC migration by conditioned PDAC cell medium and PDGF requires K_Ca_3.1 channel activity (**A**, **B**) Trajectories of migrating RLT-PSCs normalized to common starting points in the absence and presence of the K_Ca_3.1 channel blocker TRAM-34 (10 μmol/l). The radii of the grey circles depict the mean translocation of each cell population. (**C**) Summary of the migration experiments shown in A and B. # denotes statistically significant difference from unstimulated cells, and * indicates a significant effect of K_Ca_3.1 channel blockade with TRAM-34 (*p* < 0.05; *n* = 20 cells from N ≥ 3 experiments). (**D**, **E**) PDGF stimulates migration of RLT-PSCs in a K_Ca_3.1 channel dependent manner. D. Speed of migration. E. Translocation. * denotes statistically significant difference from unstimulated cells. *n* ≥ 47 cells from *N* ≥ 5 experiments.

PSCs are stimulated in a paracrine way by neighboring PDAC cells. We mimicked this situation *in vitro* by exposing RLT-PSCs to the supernatant of different PDAC cell lines. While the supernatant of BxPC3 cells does not increase motility of RLT-PSCs, those from Panc-1 and Colo357 cells induce a marked activation of RLT-PSC migration. Panel A of Figure 2 shows the trajectories of individual RLT-PSCs without stimulation (top) and after stimulation with the supernatant of Panc-1 cells (middle) or Colo357 cells (bottom). The length of the trajectories of stimulated cells is much longer than under control conditions. This is particularly evident when RLT-PSCs are treated with supernatant of Colo357 cells. Panel B of Figure 2 depicts the trajectories of RLT-PSCs treated with the K_Ca_3.1 channel inhibitor TRAM-34 (10 μmol/l). We used this high concentration since protein binding of TRAM-34 was found to be 98% [30]. TRAM-34 efficiently prevents the stimulation of migration while it has only a minor effect on basal, unstimulated migration. The experiments are summarized in panel C. When compared with unstimulated cells, the supernatant of Colo357 cells more than doubles the speed and translocation (0.45 ± 0.04 μm/min and 48.8 ± 10.2 μm versus 0.98 ± 0.09 μm/min and 110.7 ± 16.1 μm). The stimulation is largely reversed by blocking K_Ca_3.1 channels with TRAM-34 (69.9 ± 10.1 μm). We also observed a stimulatory effect on migration depending on K_Ca_3.1 channel activity when RLT-PSCs were treated with PDGF (50 ng/ml) which is expressed by PDAC cells [6] (see Figure 2D, 2E). It is noteworthy that under all conditions K_Ca_3.1 channel blockade caused a decrease of the cellular directionality by ~20%.

**Figure 3:**
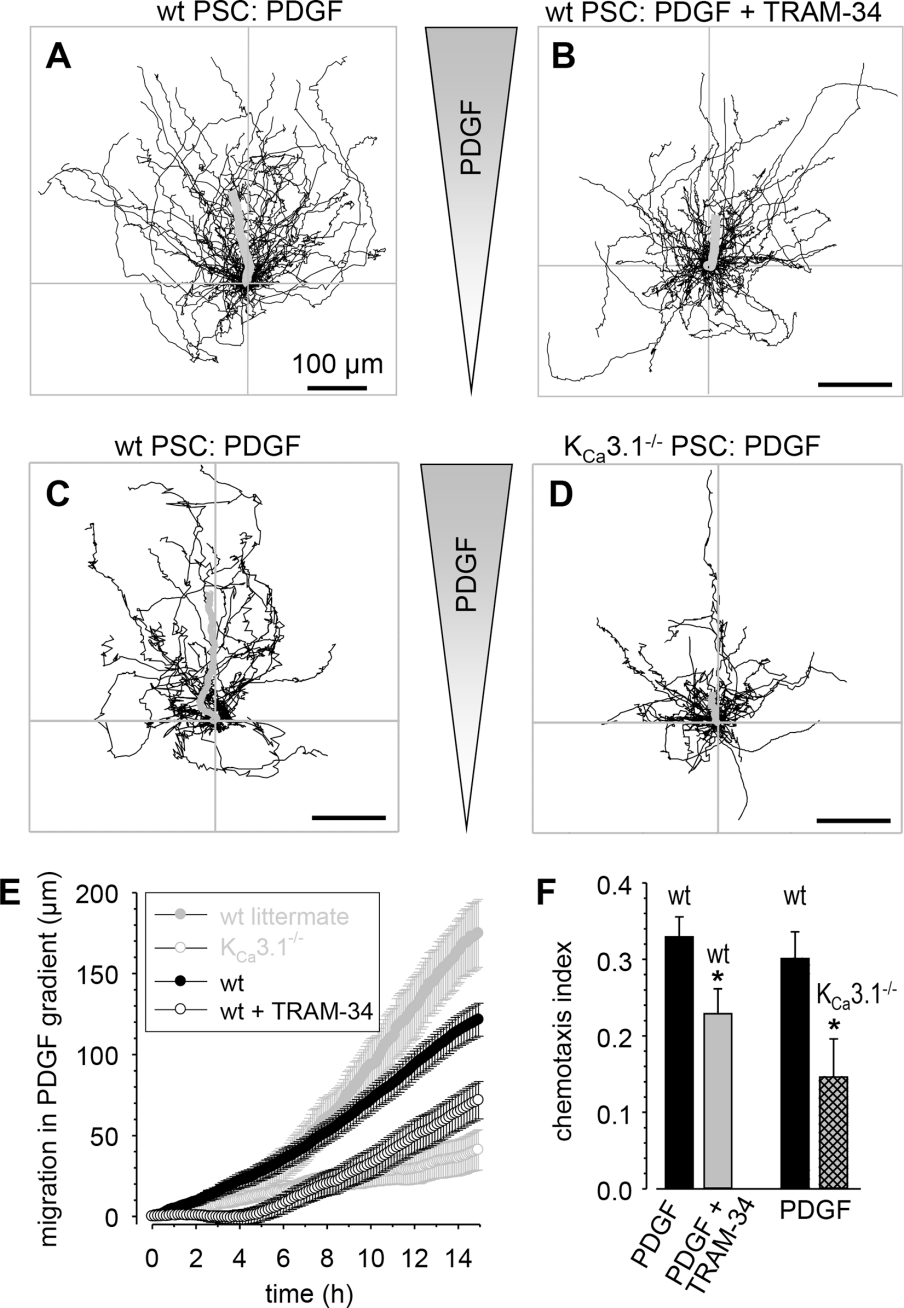
Chemotaxis of primary murine stellate cells towards PDGF is attenuated by K_Ca_3.1 channel inhibition (**A**–**D**) Trajectories of individual PSCs normalized to common starting points. The grey shaded triangle indicates the chemotactic PDGF gradient with an increasing concentration towards the top. The bold grey lines represent the average movement of the entire cell populations. (A, B) PSCs from wt mice in the absence and presence of the K_Ca_3.1 channel blocker TRAM-34. (C, D) PSCs from wt mice and their K_Ca_3.1^−/−^ littermates. (**E**) Net movement into the direction of the chemotactic gradient. K_Ca_3.1 channel blockade and K_Ca_3.1 knock-out lead to a delay and an attenuation of the chemotactic movement. (**F**) Summary of the chemotactic indices of the experiments shown in (A–D) The left bars compare the mean chemotactic index of wt PSCs in the absence and presence of the K_Ca_3.1 channel inhibitor TRAM-34. The right columns summarize the experiments with K_Ca_3.1^−/−^ PSCs compared with the respective wt littermates. *n* = 22–43 cells from *N* = 3–4 mice.

To rule out that the observed effects of K_Ca_3.1 channels on migration of RLT-PSCs are a consequence of their immortalization we performed additional experiments with primary murine PSCs. Having seen an effect of K_Ca_3.1 channel blockade on the directionality we now studied the effect of TRAM-34 or of K_Ca_3.1 channel knock-out on chemotaxis of murine PSCs towards PDGF. Murine PSCs migrate more slowly than RLT-PSCs (~8.4 μm/h versus ~16 μm/h). PDGF, however, also leads to an acceleration of murine wtPSCs which then cover ~10.5 μm/h. Murine wtPSCs chemotax efficiently in a gradient of PDGF as indicated by the asymmetric trajectories depicted in Figure 3A and 3C. The average chemotactic index is 0.33 ± 0.03 in a gradient of PDGF with peak values of up to 0.42 ± 0.03 during the second half of the experiment (Figure 3E and 3F). K_Ca_3.1 channel blockade with 10 μmol/l TRAM-34 slows down PDGF-stimulated murine PSCs to ~8.8 μm/h and causes a marked delay in the onset as well as an attenuation of chemotaxis (chemotaxis index 0.23 ± 0.03 with peak values of 0.33 ± 0.04 during the second half of the experiment). The role of K_Ca_3.1 channels in murine PSC migration and chemotaxis is further confirmed by studying K_Ca_3.1^−/−^ PSCs (Figure 3E). Both, the rate of migration (~5.8 μm/h versus ~13.5 μm/h) and the chemotaxis index (0.15 ± 0.05 versus 0.3 ± 0.3; Figure 3F) are markedly lower in K_Ca_3.1^−/−^ PSCs than in cells from the respective wildtype littermates.

### Impact of K_Ca_3.1 channels on [Ca^2+^]_i_ of RLT-PSCs

**Figure 4:**
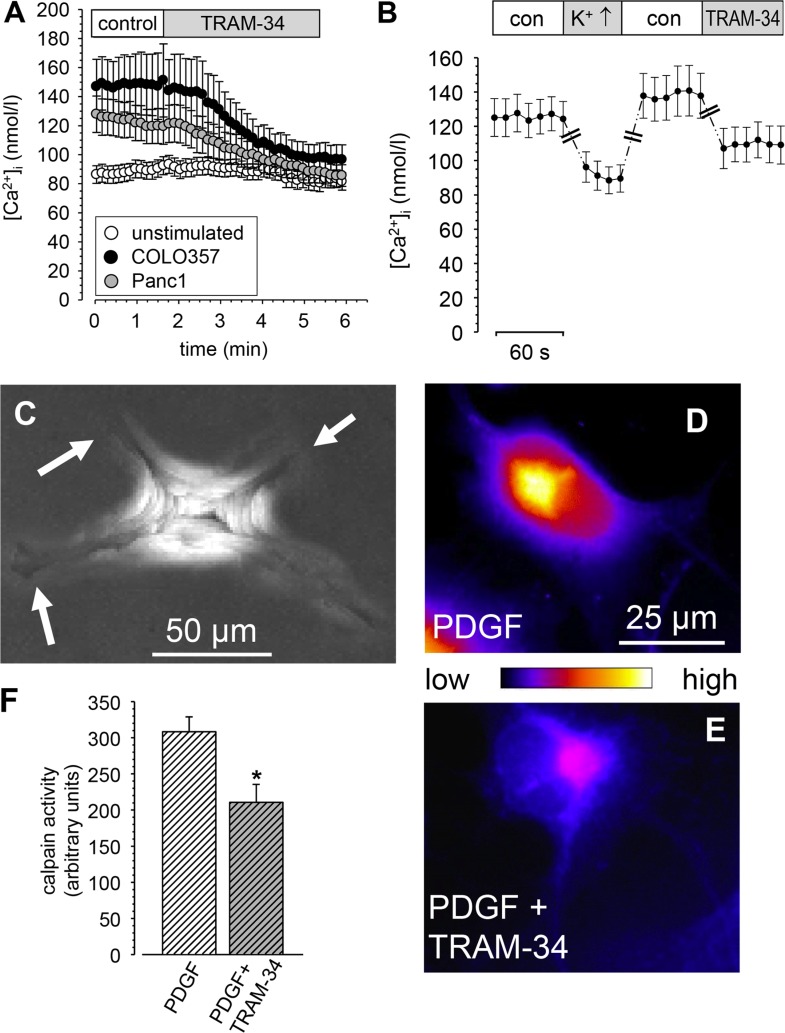
(**A, B**) Impact of K_Ca_3.1 channels on the [Ca^2+^]_i_ of RLT-PSCs stimulated with supernatant from Colo357 PDAC cells. (A) TRAM-34 (10 μmol/l) reduces the [Ca^2+^]_i_ of RLT-PSCs stimulated with the supernatants from COLO357 or Panc1 cells (*n* = 30 cells from *N* = 6 experiments each), but has no effect on untreated cells (*n* = 30 cells from *N* = 4 experiments). B. Depolarizing the cell membrane potential with 50 mmol/l KCl has a similar impact on the [Ca^2+^]_i_ of activated RLT-PSCs as blocking K_Ca_3.1 channels with 500 nmol/l TRAM-34 -(*n* = 14 cells from *N* = 5 experiments; we only plotted the steady state values of the last 30–60 s of each condition). (**C**–**F**) Blocking K_Ca_3.1 channels impairs deadhesion of RLT-PSCs by reducing calpain activity. (C) “Z-stack” of 37 images acquired in 5 min intervals of a RLT-PSC treated with PDGF and TRAM-34. The lamellipodial processes (white arrows) are clearly visible indicating that they hardly moved throughout the experiment. (D, E) Fluorescence of the calpain substrate CMAC, t-BOC-Leu-Met in RLT-PSCs treated with PDGF (D) or PDGF and TRAM-34 (E, F) Summary of *N* = 7 experiments with n ≥ 30 cells.

We next determined mechanisms by which K_Ca_3.1 channel blockade impairs migration of PSCs. We first tested whether the pretreatment of RLT-PSCs with the supernatant of Colo357 cells induced an increase of the [Ca^2+^]_i_. As shown in Figure 4A the basal [Ca^2+^]_i_ is higher in pretreated cells. The [Ca^2+^]_i_ of unstimulated cells amounts to 90.2 ± 6.1 nmol/l. It rises to 148.3 ± 21.4 nmol/l when cells are exposed for three hours to the Colo357 cell supernatant. Application of the K_Ca_3.1 channel blocker TRAM-34 (10 μmol/l) induces a decrease of [Ca^2+^]_i_ to 95.6 ± 10.2 nmol/l. The corresponding values for RLT-PSCs stimulated with the supernatant of Panc-1 cells are 109.1 ± 9.8 nmol/l and 87.0 ± 8.2 nmol/l, respectively. In unstimulated cells TRAM-34 does not induce a change of [Ca^2+^]_i_ which remains stable at 87.7 ± 6.0 nmol/l.

In another set of experiments we also studied the effect of depolarizing the cell membrane potential with a Ringer's solution containing 50 mmol/l KCl. In RLT-PSCs stimulated with Colo357 supernatant [Ca^2+^]_i_ falls to a similar extent (−34.1 ± 6.7 nmol/l) as with 500 nmol/l TRAM-34 (−27.8 ± 5.5 nmol/l; Figure 4B). Collectively, these experiments suggest that K_Ca_3.1 channels regulate [Ca^2+^]_i_ of RLT-PSCs by keeping the cell membrane potential at a hyperpolarized value. These data are consistent with an earlier study proposing the absence of voltage-gated Ca^2+^ channels in PSCs [31].

### K_Ca_3.1 channel blockade reduces calpain activity in RLT-PSCs

K_Ca_3.1 channel blockade induced a migratory phenotype suggestive of impaired detachment. Such an example is shown in Figure 4C. Here we superimposed all 37 images of a migration experiment. Four cell processes are clearly visible. The cell center is somewhat out of focus indicating that the cell body moved back and forth, whereas the peripheral processes remained attached to the substratum. Accordingly, the number of lamellipodia rises when PDGF-stimulated RLT-PSCs are treated with the K_Ca_3.1 channel blocker TRAM-34 (4.1 ± 1.0 versus 6.5 ± 1.0).

One of the Ca^2+^-dependent regulators of focal adhesion turnover is calpain. We therefore tested whether the decrease of the [Ca^2+^]_i_ following K_Ca_3.1 channel blockade is accompanied by decreased calpain activity. The results of these experiments are shown in Figure 4D–4F. Using the fluorescent calpain substrate, CMAC, t-BOC-Leu-Met, we found that calpain activity is reduced after treatment with the K_Ca_3.1 blocker TRAM-34 by ~30%.

### TRPC3 channels provide K_Ca_3.1 channels with Ca^2+^

**Figure 5:**
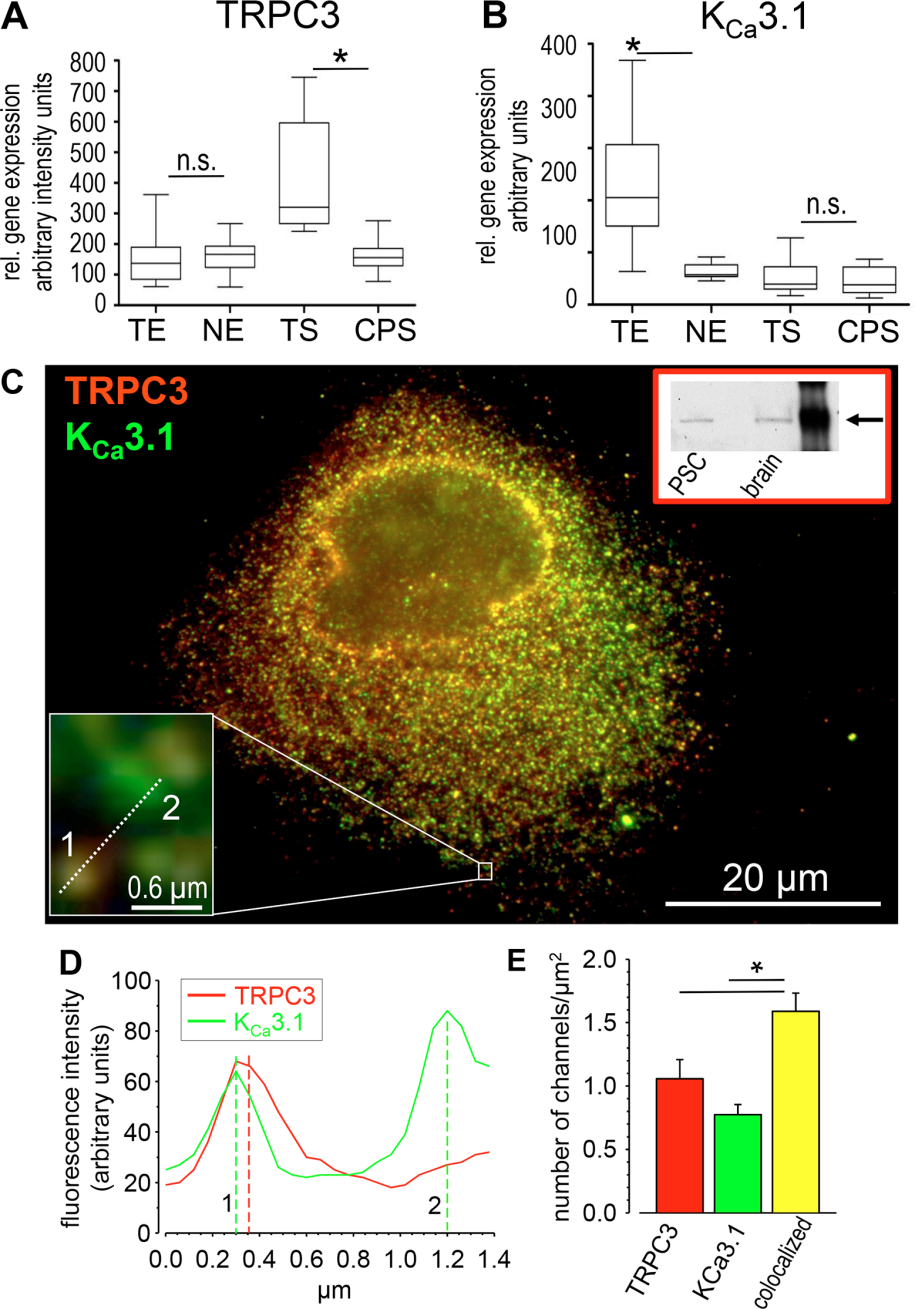
“Data mining” of published microarray analysis from microdissected freshly frozen tissue samples from PDAC (*n* = 14), normal pancreatic tissue (*n* = 11) and chronic pancreatitis specimens (*n* = 9) [56, 57] for TRPC3 (**A**) and K_Ca_3.1 channel (**B**) expression. TRPC3 expression is higher in tumor stroma (TS) than in chronic pancreatitis stroma (CPS), and that of K_Ca_3.1 channels is higher in tumor epithelium (TE) than in normal epithelial cells (NE). The Cel Files obtained from the Affymetrix MAS 5.0 software were loaded into dChip 1.3 (http://www.dchip.org), then normalized, and expression values as well as absolute calls were calculated using the PM/MM model. The analysis was carried out by using R-software, performing Wilcoxon-Mann-Whitney-Test and filtered by *p*-value (< 0.05) with Bonferroni's correction method. n.s.: not significant. (**C**–**E**) TRPC3 und K_Ca_3.1 channels are colocalised in RLT-PSCs. The Western blot verifies the TRPC3 antibody specificity (inset in C). (C) Co-staining of TRPC3 channels (red) and K_Ca_3.1 channels (green). The inset (lower left) shows the boxed area in high magnification. (D) Intensity profile along the dotted line in the inset of (C). The fluorescent spot “1” consists of colocalized TRPC3 and K_Ca_3.1 channels while “2” is a K_Ca_3.1 channel only. (E) Summary of the quantitative analysis. Approximately two thirds of the channel proteins are colocalized (*n* = 30 cells from *N* = 3 experiments).

Data mining of published microarray analyses of microdissected patient tissue samples revealed that TRPC3 channels are upregulated in the stromal compartment of PDAC as compared to that of chronic pancreatitis (Figure 5A). In contrast, K_Ca_3.1 channel expression is increased in PDAC cells but not in the tumor stroma (Figure 5B). We therefore tested whether TRPC3 channels are also expressed in RLT-PSCs. As shown by Western blot and immunofluorescence this is the case (Figure 5C). They have a similar distribution pattern as K_Ca_3.1 channels. We therefore reasoned whether TRPC3 channels might cooperate with K_Ca_3.1 channels in providing them with Ca^2+^ ions required for their activation. Such a coupling between TRPC3 and K_Ca_1.1 channels has been found in podocytes [32]. To this end, we performed co-immunostaining of TRPC3 and K_Ca_3.1 channels in RLT-PSCs. Figure 5C–5E reveal that approximately two thirds of TRPC3 and K_Ca_3.1 channels are colocalized.

**Figure 6:**
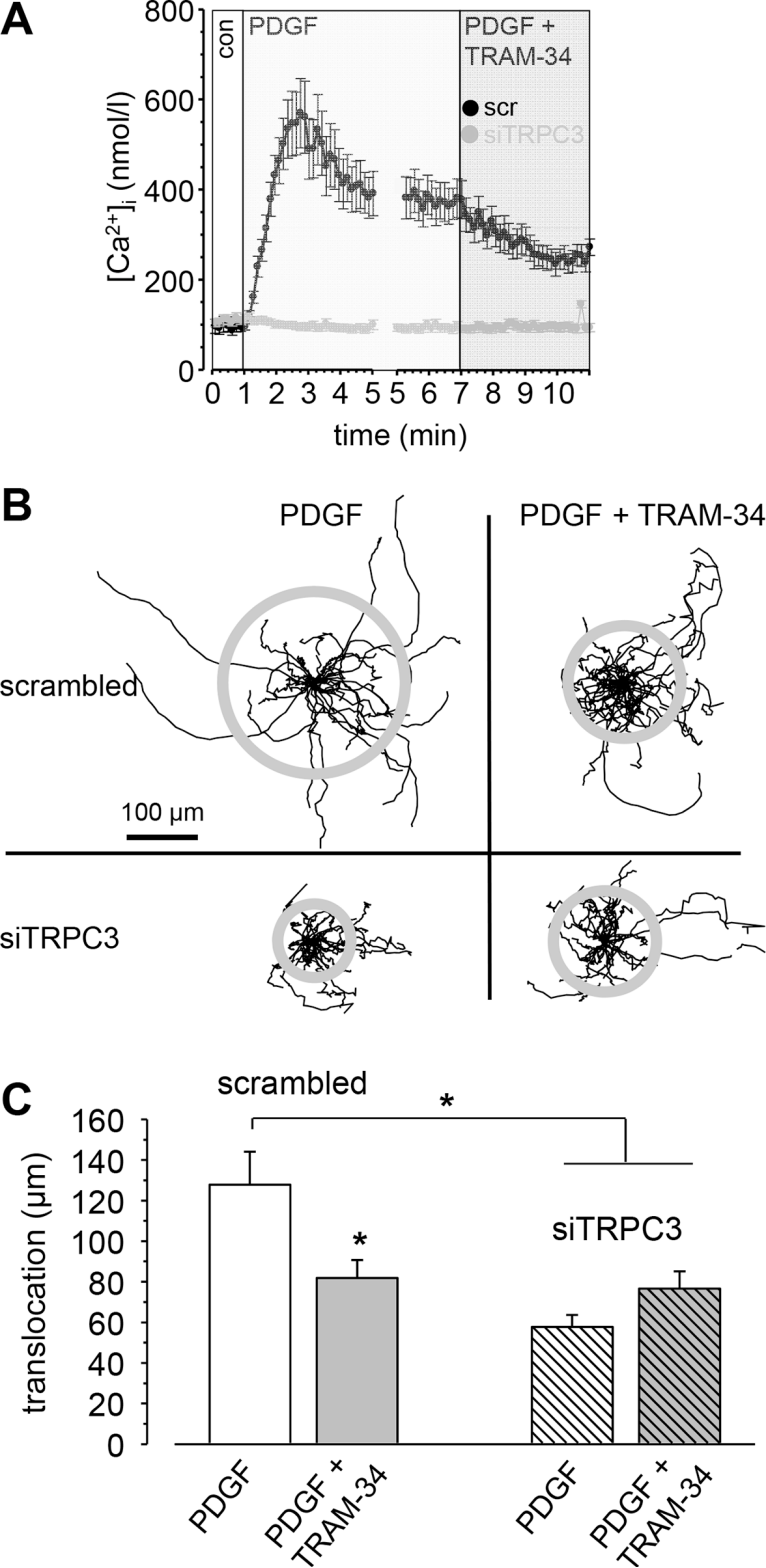
Impact of TRPC3 channels on the [Ca^2+^]_i_ and migration of RLT-PSCs (**A**) PDGF induces a transient rise of the [Ca^2+^]_i_. Inhibition of K_Ca_3.1 channels with TRAM-34 leads to a reduction of the [Ca^2+^]_i_ (*n* = 19 from *N* = 3 experiments). Knockdown of TRPC3 channels abrogates the increase of the [Ca^2+^]_i_ following stimulation with PDGF (*n* = 41 from *N* = 3 experiments). (**B**) Trajectories of RLT-PSCs transfected with scrambled siRNA or with siTRPC3 RNA. The trajectories are normalized to common starting points, and the radii of the grey circles represent the mean translocation of the cell populations. (**C**) Summary of the migration experiments. siTRPC3 transfection strongly reduced the translocation and makes the cells unresponsive to the K_Ca_3.1 channel blocker TRAM-34. *n* ≥ 31 cells from *N* ≥ 3 experiments.

Next we evaluated their role in regulating the [Ca^2+^]_i_ of RLT-PSCs (see Figure 6A). [Ca^2+^]_i_ rises transiently to ~550 nmol/l when stimulated with 50 ng/ml PDGF. After 5 min [Ca^2+^]_i_ has gradually fallen to ~400 nmol/l. Blocking K_Ca_3.1 channels with TRAM-34 in the continued presence of PDGF then leads to a decrease of [Ca^2+^]_i_ to ~250 nmol/l. In siTRPC3-RLT-PSCs PDGF fails to elicit an increase of [Ca^2+^]_i_.

### TRPC3 channels in PDGF-stimulated migration of RLT-PSCs

We finally investigated whether TRPC3 channels are involved in the stimulation of RLT-PSC migration. We compared migration of PDGF-stimulated siTRPC3-RLT-PSCs with that of control RLT-PSCs transfected with scrambled siRNA (Figure 6B and 6C). Control cells cover much larger distances (127.8 ± 16.2 μm) than siTRPC3 cells (57.7 ± 5.6 μm). Blocking K_Ca_3.1 channels with TRAM-34 has no additional inhibitory effect, which is consistent with the notion that TRPC3 channels are functionally cooperating with K_Ca_3.1 channels by providing them with Ca^2+^.

## DISCUSSION

This study is one of the first to explore the expression and function of ion channels in pancreatic stellate cells. The main findings of our study are that (i) pancreatic stellate cells functionally express K_Ca_3.1 channels whose activity (ii) is required for (directed) migration by (iii) regulating the [Ca^2+^]_i_ and thereby (iv) calpain activity. Finally, we showed that (v) the role of K_Ca_3.1 channels in cell migration depends on cooperation with TRPC3 channels which are upregulated in the stroma of PDAC tissue samples. Thus, K_Ca_3.1 channels are as expected closely linked to the intracellular Ca^2+^ homeostasis of PSCs.

So far, only few studies addressed the role of the [Ca^2+^]_i_ in PSC function such as proliferation and the molecular nature of the channels involved in Ca^2+^ signaling [20, 31]. They showed that (nuclear) Ca^2+^ signals are an important determinant of the proliferation of activated PSCs and that the “Ca^2+^ signature” of activated PSCs differs from that of quiescent PSCs [31]. However, several studies performed with hepatic stellate cells (HSCs), close “relatives” of PSCs, point to a causal relationship between intracellular Ca^2+^ signaling and pro-inflammatory as well as profibrotic events. Thus, norepinephrine-induced Ca^2+^ oscillations were linked to enhanced cytokine secretion (e.g. CCL5 (RANTES), IL-8) and thereby autocrine activation of HSCs [33]. Their activation by angiotensin II relies on more prolonged elevations of the [Ca^2+^]_i_ that in turn also involves the CD38-dependent production of the intracellular messengers cyclic ADP-ribose (cADPR) and nicotinic acid adenine dinucleotide phosphate (NAADP). This is a pathophysiologically relevant mechanism since CD38^−/−^ mice are protected to some extent from liver fibrosis induced by bile duct ligation [34]. Finally, K_Ca_3.1 inhibition was shown to exert antifibrotic effects that could be related to an inhibition of TGF-β signaling in hepatic stellate cells [24].

To our knowledge there are only very few studies showing a direct role of TRP channels in PSC function. TRPC1 channels are contributing to the pressure-induced activation of PSCs as revealed by a TRPC1-dependent stimulation of migration and Ca^2+^ influx [22]. TRPA1 channels are involved in the development of acute and chronic pancreatitis. Since they were rather found in pancreatic nerve fibers and dorsal root ganglia neurons innervating the pancreas than in PSCs, these channels were proposed to mediate a neurogenic inflammation of the pancreas [35, 36]. Thus, in this model TRPA1 channels were thought to exert an indirect effect on pancreatic stellate cells. TRPV4 channels are functional in PSCs [19]. However, the fact that TRPV4^−/−^ mice developed the same degree of (acute) pancreatitis as wildtype mice argues against a role of TRPV4 channels in this disease. Our findings could point to a role of TRPC3 channels in pancreatitis. The attenuation of an acute pancreatitis in TRPC3^−/−^ animals was explained on the basis of an altered exocytotic protease secretion from acinar cells. In contrast to wt cells, exocytosis was maintained in TRPC3^−/−^ acinar cells during supramaximal stimulation thereby preventing their pathological intracellular activation of proteases [32]. Since PSCs also become activated in an acute pancreatitis [37] our findings raise the possibility that a diminished activation of PSCs might have also contributed to the attenuated acute pancreatitis in TRPC3^−/−^ mice [38]. Along these lines it was also suggested that bradykinin-mediated Ca^2+^ signaling in PSCs could contribute to causing an ACE blocker-induced acute pancreatitis. These drugs inhibit bradykinin breakdown and could thereby activate PSCs by augmenting intracellular Ca^2+^ [39]. Collectively, these studies show that Ca^2+^ signaling plays an important role in stellate cell activation and hence qualifies the involved Ca^2+^ (sensitive) transport proteins as potential targets in stellate cell-directed antifibrotic and antiinflammatory therapies [40, 41]. However, so far there is no direct evidence for a role of ion channels in PSCs in the context of PDAC.

We found that K_Ca_3.1 and TRPC3 channels play important roles in stimulated migration of PSCs. Previously, we suggested that ion channels like K_Ca_3.1 are part of the migratory machinery by inducing localized changes of cell volume (reviewed in [27]). This hydrodynamic model of cell migration was confirmed by other groups [42–44]. Here, we report an additional mechanism by which K_Ca_3.1 channels can affect cell migration, namely by cooperating with TRPC3 channels. We found in PSCs that K_Ca_3.1 and TRPC3 channels are closely colocalized in the plasma membrane so that Ca^2+^ entering the cell via TRPC3 channels can activate neighboring K_Ca_3.1 channels. The ensuing hyperpolarization of the cell membrane potential increases or maintains the electrochemical driving force for Ca^2+^ entry. Conversely, a depolarization of the cell membrane potential by either blocking K_Ca_3.1 channels or by exposing the cell to 50 mmol/l KCl leads to a decrease of the [Ca^2+^]_i_. An excessive positive feedback between K_Ca_3.1 activation induced by TRPC3-mediated Ca^2+^ influx may be prevented by the depolarizing impact of cation influx through TRPC3 channels. Alternatively, it is conceivable that the increase of the [Ca^2+^]_i_ also activates other ion channels that depolarize the cell membrane potential such as TRPM4 channels or members of the ANO/TMEM16 family of Cl^−^ channels [45, 46]. However, because of the limited knowledge of the PSC transportome these two latter theories await experimental verification.

Led by the observation that K_Ca_3.1 channel blockade results in impaired deadhesion of PSCs we studied the role of the Ca^2+^ sensitive protease calpain which is a regulator of focal adhesions [47]. In T lymphocytes inhibition or knockdown of calpain induced a similar migratory phenotype as in PSCs treated with the K_Ca_3.1 channel blocker TRAM-34. The detachment of the trailing edge was disturbed [47]. So far we do not know whether the impaired directionality and chemotaxis following K_Ca_3.1 channel inhibition is also due to reduced calpain activity. In neutrophils calpain is involved in chemotaxis [48].

**Figure 7:**
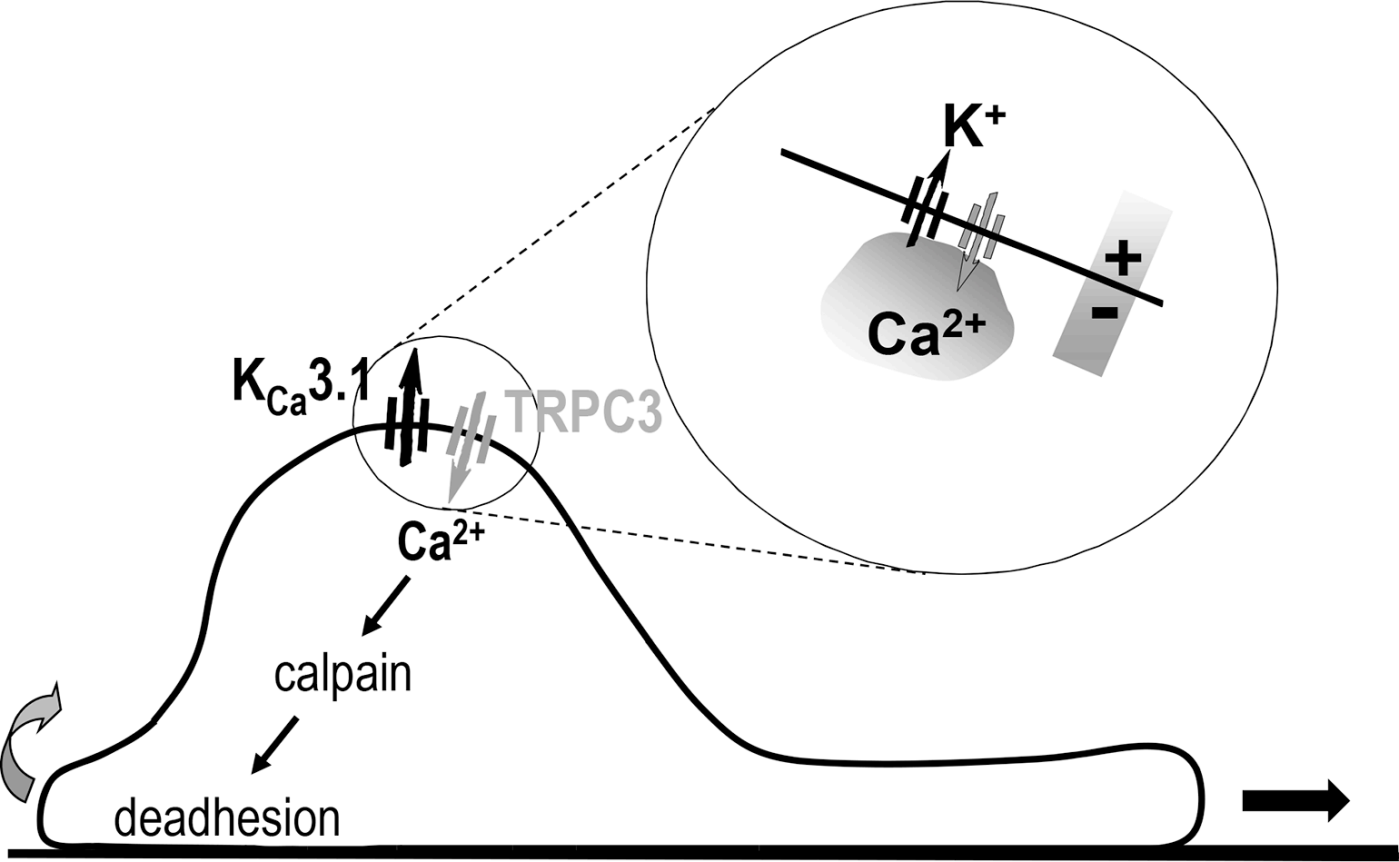
Summary scheme of how K_Ca_3.1 and TRPC3 channels cooperate with each other in order to support migration K_Ca_3.1 channels provide electrochemical driving force for Ca^2+^ entry via TRPC3 channels. [Ca^2+^]_i_ in turn maintains activity of K_Ca_3.1 channels and, among others, activates calpain thereby promoting deadhesion of migrating stellate cells.

The cooperation between K_Ca_3.1 and TRPC3 channels is reminiscent to findings made in rat mesenteric artery endothelial cells where a similar functional interaction between TRPC3 and K_Ca_3.1 channels was found [49]. In migrating glioma cells TRPC1 and ClC-3 channels form a functional unit colocalized in caveolae. The role of ClC-3 channels in migration and chemotaxis depends on their supply with Ca^2+^ ions via TRPC1 channels [50]. Our results suggest that K_Ca_3.1 channels “utilize” Ca^2+^ ions entering the cell via TRPC3 channels. In Figure 7 we present a model of the cooperation between K_Ca_3.1 and TRPC3 channels in PSC migration. Our findings help to better understand the physiology of PSCs which is important when viewed against the background of the controversial discussion on the role of PSCs in the progression of PDAC [10]. Our findings and those from other groups in hepatic stellate cells clearly show that ion channels are crucial players in PSC physiology and pathophysiology.

## MATERIALS AND METHODS

### Cell culture

Experiments were performed with a cell line of human pancreatic stellate cells, RLT-PSC [51] and three PDAC cell lines, BxPC3, Panc1, and Colo357. They were cultured in DMEM/F12 (RLT-PSC), RPMI 1640 (BxPC3), DMEM/RPMI 1640 (Colo357) and DMEM (Panc1) supplemented with 10% fetal calf serum (FCS, PAA Gold) at 37°C in a humidified air/5% CO_2_ atmosphere. PDAC cells were grown for three days to 80% confluence before conditioned medium was collected. It was cleared by sterile filtration and stored at 4°C before use.

### Isolation of primary murine pancreatic stellate cells

Procedures were approved by the local authorities. The pancreata of 8 - 12 weeks old mice (C57BL/6; Charles River, Sulzfeld, Germany) or of K_Ca_3.1^−/−^ mice and the wt littermates (SV129 background) were removed after the animals were anaesthetized with isofluran and killed by cervical dislocation. The pancreas was isolated, cut into pieces and suspended in 3 ml Grey's balanced salt solution (GBSS) containing 2.5 mg collagenase P. After shaking for 30 min at 37°C the enzyme-containing solution was further diluted with GBSS and centrifuged. The pellet was resuspended in DMEM/F-12 medium containing 10% FCS and 1% penicillin/streptomycin and plated in an FCS-coated cell culture dish. After two hours, the dish was washed with media. Since PSCs were the only cells that attached strongly [20] they could be easily isolated from the other non-adherent cells. Primary murine PSCs were used for the experiments after passaging them 1 to 2 times. The identity of primary murine PSCs was revealed by the disappearance of vitamin A droplets and the appearance of α-smooth muscle actin and glial fibrillary acidic protein after a couple of days in culture.

### TRPC3 knockdown

RLT-PSCs were grown over night in a 60 mm dish to 70% confluency. TRPC3 siRNA or control siRNA (Invitrogen; 24 pmol/l each) were mixed with Opti-MEM (Invitrogen) and siRNA Lipofectamin (Invitrogen) according to the manufacturer's instructions. We used the following double-stranded oligos: sense: 5′-GCAGCAGCUCUUGACGAUCUGGUAU-3′ antise nse: 5′-AUACCAGAUCGUCAAGAGCUGCUGC-3′ [52]. Cells were used for experiments 48 h after transient transfection. In parallel to migration experiments and measurements of [Ca^2+^]_i_ knockdown efficiency was confirmed by Western blot.

### Western blot

RLT-PSCs grown to 80% confluency were washed before lysis in 50 mmol/l Tris-HCl pH 7.6, 150 mmol/l NaCl, 10 mmol/l Nonidet-P40, 3.5 mmol/SDS, 10% Na-deoxycholate, PhosSTOP (phosphatase inhibitor cocktail; Sigma) and cOmpleteMini (protease inhibitor cocktail; Sigma). Lysates were size-fractionated with SDS-PAGE (10%) and blotted onto PVDF membranes. K_Ca_3.1 and TRPC3 channel proteins were labelled by successive incubation with the respective primary (rabbit anti-K_Ca_3.1, Sigma-Aldrich, 1.0 mg/ml, 1:2.000; rabbit anti-TRPC3, Alomone Laboratories, 0.8 mg/ml, 1:400; mouse anti-actin, Sigma-Aldrich, 30.3 mg/ml, 1:15.000) and secondary antibodies (peroxidase conjugated goat anti-rabbit and anti-mouse, Sigma-Aldrich) followed by chemiluminescent detection (Pierce).

### Immunofluorescence

Staining of K_Ca_3.1 channels was performed according to a protocol described previously [26]. Antibody titers were: primary rabbit anti-K_Ca_3.1 (1.0 mg/ml), 1 : 1.000, 1h and secondary goat anti-rabbit Alexa 488, 1 : 1.000, 1h. After staining the cells were fixed again for 10 min in 3.5% paraformaldehyde.

For double-labelling of K_Ca_3.1 and TRPC3 channels this protocol was modified because both primary antibodies were derived from rabbit. First, K_Ca_3.1 channels were stained as described above. Then the cells were fixed for 10 min in 3.5% paraformaldehyde before staining of TRPC3 channels (TRPC3 antibody concentration 0.8 mg/ml, 1:300) following the same protocol. We maximally increased the concentration of secondary antibody used to label K_Ca_3.1 channels (goat anti-rabbit Alexa 488, 1 : 200) without obtaining unspecific binding in the absence of the primary antibody. At the same time we maximally diluted the concentration of the secondary antibody used to mark TRPC3 channels (goat anti-rabbit Cy3, 1 : 800) without diminishing the TRPC3 staining density. The high concentration of goat anti-rabbit Alexa 488 secondary antibody saturated all binding sites of primary anti-K_Ca_3.1 antibodies because the subsequent incubation with goat anti-rabbit Cy3 (1 : 800) did not produce any additional labelling.

We used an inverted microscope (Axiovert 200, Zeiss, Oberkochen, Germany) equipped with a digital camera (model 9.0, RT-SE-Spot, Visitron, Puchheim, Germany) and a 100x, 1.45 oil immersion objective for documentation. Data acquisition and analysis were performed with Metavue software (Visitron). The average density and colocalization of K_Ca_3.1 and TRPC3 channels were determined in 6 areas (3 μm x 3 μm) per cell. After correcting pixel shifts of superimposed images, we used the linescan tool in order to determine the optical center of the fluorescent spots. We assumed a colocalization between K_Ca_3.1 and TRPC3 channels when the distance between their optical centers was less than 1 pixel (corresponding to 60 nm)[53].

### Patch clamp experiments

Functional expression of K_Ca_3.1 channels was revealed by performing the patch clamp experiments using the whole cell configuration as described previously [29]. Experiments were performed at room temperature. The holding potential was −40 mV which is close to the cell membrane potential. Current amplitude was determined at the end of the depolarizing ramp protocol (5 s; −84 to +56 mV). The extracellular solution was composed of (in mmol/l): 140 NaCl, 5 KCl, 10 HEPES, 1 MgCl_2_, and 1 CaCl_2_, pH 7.4 with NaOH. The intracellular solution contained (in mmol/l): 140 KCl, 10 HEPES, 1.3 EGTA, 1.217 CaCl_2_, and 1 MgCl_2_, pH 7.4 with KOH. The calculated free Ca^2+^ concentration of the internal solution was 1 μmol/l. Clotrimazole (1 μmol/l) and 1-ethyl-2-benzimidazolone (1-EBIO; 50 μmol/l) were dissolved in DMSO, added to the extracellular solution, and applied for a time period of 2 and 10 min, respectively. The final DMSO concentration was < 0.1%. Recordings were analyzed using FitMaster and Origin 7.5 software.

### Migration experiments

Migration of PSCs was quantified by means of live-cell imaging as described previously [28]. Cells were seeded 3 h prior to the experiment into tissue culture flasks coated with a “basement membrane-like matrix” (Table 1). Image acquisition in 5 min intervals was controlled by HiPic or WASABI software (Hamamatsu). Migration was quantified as the movement of the cell center with time. We calculated the cell speed (in μm/min) as a three-point difference quotient and the translocation (in μm) as the net distance covered within the experiment. The directionality was derived from the quotient of translocation and total path length.

**Table 1:** Composition of the extracellular matrices

	basement membrane matrix
RPMI 5×	10.4 g/L
HEPES 5×	10 mmol/L
NaOH	~15 μL 1M NaOH per 1 mL matrix solution [58]
laminin	100 μg/mL
fibronectin	100 μg/mL
collagen IV	1 mg/μL
H_2_O	ad 1 mL

### Chemotaxis experiments

Chemotaxis experiments were performed in chemotaxis chambers (ibidi, Martinsried, Germany) coated for one hour with a “basement membrane”-like matrix (see Table 1). Cells were seeded in HEPES-buffered RPMI at a density of 8 × 10^5^ cells/ml and allowed to settle for three hours. Thereafter, the medium was exchanged and if applicable supplemented with 10 μmol/l TRAM-34. A chemotactic gradient was established according to the manufacturer's instructions by adding 17 μl of PDGF-containing medium (400 ng/ml) to one of the reservoirs of the chemotactic chamber. Chemotaxis was monitored by means of live-cell imaging for ~16 h in 8 min intervals. The migratory behavior was analysed by manually tracking the cell center of each cell. Speed and translocation were calculated as described above. Chemotactic efficiency was determined as the chemotaxis index (CI) which is the ratio of net movement into the direction of the gradient and the total distance covered during the course of the experiment. Movement towards increasing concentrations of PDGF is indicated by a positive CI.

### Measurements of the intracellular Ca^2+^ concentration

We determined the intracellular Ca^2+^ concentration ([Ca^2+^]_i_) ratiometrically with the fluorescent Ca^2+^ indicator fura-2 as described previously [54]. Cells were plated on glass bottom dishes (Willco, Amsterdam, The Netherlands) coated with a basement membrane-like matrix and pretreated in the same way as for migration experiments. 3 h after plating, cells were loaded with fura2-AM (3 μmol/l, Calbiochem) for 20 min at 37°C in the respective culture medium. Then the cells were transferred to the stage of an inverted microscope (Axiovert 200, Zeiss) and continuously superfused with prewarmed (37°C) Ringer's solution of the following composition (in mmol/l): 122.5 NaCl, 5.4 KCl, 1.2 CaCl_2_, 0.8 MgCl_2_, 10 HEPES, 5.5 glucose, pH7.4. When indicated the extracellular K^+^ concentration was elevated to 50 mmol/l – isosmotically replacing 44.6 mmol/l NaCl. The mean cellular fluorescence intensities were corrected by background subtraction and measured in 10 s intervals. Ca^2+^ measurements were calibrated at the end of each experiment. Maximal and minimal ratios were determined separately for each cell following the application of Ringer's solutions containing ionomycin (1 μM) and either 5 mM EGTA or 5 mM Ca^2+^.

### Determination of calpain activity

To determine calpain activity in single cells the Boc assay was used [55]. Glass bottom dishes (Greiner Bio-One, Frickenhausen, Germany) were coated for 30 min with 1:10 diluted basal membrane-like matrix, before ~2.3 × 10^4^ cells were plated in growth medium to attach for 3–4 hours at 37°C and 5% CO_2_, 95% air. 5 min prior to the experiment DMEM/F12 medium was replaced with Hepes-buffered Ringer solution. Cells were then either treated in the presence or absence of 50 ng/ml PDGF with DMSO (1:1000) or TRAM-34 (10 μmol/l) for 30 min. Thereafter, cells were transferred to the microscope stage (Axiovert 200) and the calpain substrate 7-amino-4-chloromethylcoumarin, t-BOC-L-leucyl-L-methionine amide (CMAC, t-BOC-Leu-Met; 10 μM; Molecular Probes) was added. After 20 min at 37°C fluorescence images were taken using a digital camera (model 9.0, RT-SE-Spot, Visitron) and the MetaVue software (Visitron). We used the following filter set: excitation 365/12 nm, beam splitter 395 nm, emission 397 nm. Image exposure settings were identical within each experiment: 100 msec for CMAC, t-BOC-Leu-Met. Fluorescence intensity was measured over the entire projected cell area and corrected for background fluorescence in ImageJ.

### Statistics

All data are presented as means ± SEM. Statistically significant differences were determined with paired or unpaired *t*-test or with one-way ANOVA and post hoc Bonferroni test for multiple comparisons as appropriate. For non-parametric comparison of two groups, we applied Wilcoxon-Mann-Whitney-Test.

## Abbreviations

CCL5: C-C motif chemokine ligand 3
K_Ca_3.1: Ca^2+^-activated potassium channel of intermediate conductance
PDGF: platelet-derived growth factor
PSC: pancreatic stellate cell
RANTES: regulated upon activation normal T cell expressed and secreted
TRAM-34: 1-[(2-chlorophenyl)-di(phenyl)methyl]pyrazole (K_Ca_3.1 channel blocker)
TRPC1 and TRPC3: transient receptor potential cation channel, subfamily C (canonical), member 1 and 3
TRPV4: transient receptor potential cation channel, subfamily V (vanailloid) member 4
TRPA1: transient receptor potential cation channel, subfamily A (ankyrin), member 1
